# Incidence and Characteristics of Injuries during the 2010 FELDA/FAM National Futsal League in Malaysia

**DOI:** 10.1371/journal.pone.0095158

**Published:** 2014-04-14

**Authors:** Mohamad Shariff A. Hamid, Zulkarnain Jaafar, Azril Syazwan Mohd Ali

**Affiliations:** 1 Unit of Sports Medicine, Faculty of Medicine, University of Malaya, Kuala Lumpur, Malaysia; 2 Department of Orthopaedic, Hospital Raja Perempuan Zainab II, Jalan Hospital, Kota Bahru, Kelantan, Malaysia; University of Eastern Finland, Finland

## Abstract

**Objective:**

In Malaysia, futsal is a popular sport played by individuals across all ages and genders. Despite its popularity, information on futsal related injury in Malaysia is not available. The purpose of this study is to examine the injury incidence and injury patterns among amateur men and women futsal players in Malaysia.

**Methods:**

Players reported injury to the tournament medical team during the FELDA/FAM National Futsal League 2010 were interviewed and assessed by a Sports Medicine registrar. Player's socio-demographic profiles and information about the injury were documented in the injury report form adapted from medical report form used by FIFA: Medical Assessment and Research Centre (F-Marc).

**Results:**

A total of 86 injuries were reported from 141 matches, equivalent to an incidence of 91.5 injuries per 1000 player hours (95% CI 72.2 to 110.8), or 61.0 injuries per 1000 player matches (95% CI 48.1 to 73.9). Most were minor injuries resulted from contact with another player. Injuries often involved the lower extremity (44%) followed by the trunk (14%) and the upper limb (13%). Ankle (n = 7; 39%) and knee (n = 6; 33%) sprains were the most prevalent diagnoses of time-loss injuries. A significant association between time-loss and type of injury was found *χ* ^2^ (1,N = 86) = 3.99, *p* = 0.04. In addition, time-loss injury was significantly associated with playing surface *χ* ^2^ (1,N = 86) = 10.11, *p* = 0.018.

**Conclusion:**

The injury rate during the FELDA/FAM National Amateur Futsal Men's League in Malaysia was lower compared with previous Futsal World Cups competition. Most injuries resulted from contact with another player were minor and did not lead to time-loss from participation. Time-loss injury was significantly associated with type of injury and playing surface.

## Introduction

Futsal is a variant of football played indoors on a smaller pitch. It started in South America in 1930 and has since become a popular indoor sport worldwide. Currently there are around one million registered futsal players all over the world [1]. Futsal is a team sport characterized as an intermittent high-intensity strenuous sport [2], [3]. The game relies on individual skills and creativity as well as team efforts to create goals.

Unlike outdoor football and indoor soccer (more common in USA and Canada), the rules and laws in futsal are stricter. Only limited-contact between players is allowed. To uphold fair plays and reduce the incidence of injury in the smaller playing area, side tackling, aggressive body contacts and robust play are not allowed [4]

As relatively a recent sport, data on futsal injuries are limited. Current literature reported higher risks of injury in futsal compared with outdoor football. The incidence of injury ranges from 168.5 to 236.0 per 1000 player hours among top-level international male futsal player [5], [6]. This difference was attributed to the nature of the game, which is associated with higher speed of play, smaller playing field, hence increasing the risk of collisions [5]. Being one of the most practiced sport in the world information on injury incidence and injury characteristics would allow better understanding and provide a foundation for necessary steps of injury prevention [7]. In Malaysia, futsal was first introduced near the end of the 20^th^ century. Following the introduction of National Futsal League in 2004, futsal became one of the most popular sports played by both men and women across all ages [8]. Despite its popularity, information on injury incidence and injury patterns among Malaysian futsal players is unavailable. The purpose of this study was to examine the incidence and characteristics of injuries during the 2010 FELDA (Federal Land Development Authority)/FAM (Football Association of Malaysia) National Futsal League in Malaysia. In addition, the incidence of time-loss injuries and factors associated were explored.

## Methods

This study was conducted during the 4^th^ FELDA/FAM National Futsal League 2010. The competition consisted of two separate men and women's leagues. Players who reported injury to the tournament medical team were eligible to take part in this study. The tournament medical team comprised a Sports Medicine registrar assisted by paramedics (medical assistant, first aiders, stretcher bearers, ambulance drivers and medical coordinator). The medical team were on duty throughout the tournament. The Sports Medicine registrar is a certified medical doctor (MD) and had at least 3 years of working experience in Sports Medicine. All players reported injury to the tournament medical team were interviewed and assessed by the same Sports Medicine registrar. Individual consent was obtained before assessment. Player's socio-demographic profiles including their date of birth, sex, ethnicity and playing experience were recorded. More importantly information about the injury were documented in the injury report form available as Figure S1.

Injured players were asked to present themselves to the medical area and complete the injury report forms as soon as match ended. Data collection procedures followed the consensus statement on injury data collection procedures in football [5]. Further the injury report form used was adapted from medical report form as used by FIFA (Federation Internationale de Football Association): Medical Assessment and Research Centre (F-Marc) [5].

In this study an injury was defined as “any physical complaint sustained by a player that results from football match or football training, irrespective of the need for medical attention or time loss from football activities” [9]. Recurrent injury was defined as “an injury of the same type and at the same site as an index injury and which occur after player's return to full participation from the index injury” [9].

### Ethics statement

Information about the study including study objective and procedures were explained to all participants, Participants were required to sign the informed consent form before participation. Written consents were obtained from parents or guardians and the participant if they were less than 18 years old. The Medical Ethics Committee of the University Malaya Medical Centre (Ref. no:794.10) and the Football Association of Malaysia (FAM) (Ref. no: FAM/233/16/25(10)DAA/AFD/hah) approved this study.

### Injury rates and statistical analysis

Injury rates were expressed as number of injuries per match, per 1000 player hours and per 1000 player matches [5]. The definition of player hours and player matches were based on those described by previous authors [5], [9]. Total player hours were calculated by multiplying the number of matches played by ten (number of players in the field) by 40/60 hours [9]. Extra time and reduced numbers of players in the field were not considered as they rarely occur. Injuries per 1000 player hours were calculated as: number of injuries x 1000 divided by total player hours. Total player matches were calculated by multiplying the number of matches played by ten [9]. Therefore, injuries per 1000 player matches were calculated as: number of injuries x 1000 divided total player matches. The 95% confidence intervals (CIs) of injury rates, were calculated as: incidence ±1.96 x incidence/√number of injuries [5], [9].

Data were analysed descriptively with normality test conducted using the Shapiro-Wilk test. Potential factors associated with time-loss injury were explored with Chi-square tests or Mann-Whitney U (as age and playing experience were not normally distributed). Injury rates between groups (gender, tournament stages and playing position) were compared using statistical methods as described by Lindenfeld et al. (1994) [10]. All statistical analyses were calculated using the Statistical Package for Social Science (SPSS) ver 19.0, with significance level set at *p*<0.05.

### Tournament

The 2010 FELDA/FAM National Futsal League started in July and completed in November. This competition comprised men and women's leagues. A total of 141 matches were played in the tournament (111 in the league stage and 30 in the final round). Two round matches were played on the first 2 weekends (Saturday and Sunday) of each month except in August because the fasting month of Ramadan. Seven preliminary rounds matches and a final round match were played in the tournament. Matches were played in four different venues with either parquet (Sultan Abdul Halim Indoor Stadium and Panasonic Indoor Stadium) or synthetic vinyl (Triways Sports Complex and Community Sports Complex) flooring.

## Results

A total of 469 (238 men and 230 women) players from 32 teams (16 men and 16 women teams) participated in the 4^th^ edition of FELDA/FAM National Futsal League 2010. All players, took part in the tournament were amateur players according to FIFA rules [11]. Eighty-six injuries were reported throughout the competition, covering 940 of player hours. The socio-demographic characteristics of injuries during the tournament are displayed in table 1.

**Table 1:** Socio-demographic characteristics of injured players during the 2010 FELDA/FAM National Futsal League in Malaysia.

Characteristics	Median±IQR (range)	Frequency (%)
Age (years)	24.0±IQR6.0 (14.0–44.0)	
Experience (years)	3.0±IQR3.0 (1.0–13.0)	
Sex		
Men		45 (52.3)
Women		41 (47.7)
Ethnicity		
Malay		82 (95.3)
Others		4 (4.7)
Tournament stage		
Preliminary		71 (82.6)
Final		15 (17.4)
Nature of injury		
Sprain		28 (32.6)
Strain		14 (16.3)
Contusion		31 (36.0)
Others		13 (15.1)
Injured region		
Lower limb		56 (65.1)
Upper limb		11 (12.8)
Trunk (chest & back)		12 (14.0)
Head & neck		7 (8.1)
Player's position		
Goalkeeper		22 (25.6)
Defender		18 (20.9)
Forward		46 (53.5)
Injury circumstances		
Contact		54 (63.0)
Non-contact		32 (37.0)
Injury severity		
Time loss		25 (29.1)
No time loss		63 (70.9)

Note: IQR  =  inter-quartile range; FELDA  =  Federal Land Development Authority; FAM  =  Football Association of Malaysia.

### Injury incidence

The total injury incidence was, 0.61 injuries per match (95% CI 0.5 to 0.7), 91.5 injuries per 1000 player hours (95% CI 72.2 to 110.8), or 61.0 injuries per 1000 player matches (95% CI 48.1 to 73.9) (see table 2). No significant difference in the injury rates between the league and final stages of tournament was found (95.9 vs. 75 injuries per 1000 player hours)(*Z* = 0.87, *p*>0.05). Injury incidence was slightly higher in men compared with women's leagues (96.4 vs. 86.6 per 1000 hours). The difference between genders however was not statistically significant (*Z* = 0.50, *p*>0.05).

**Table 2:** Incidence and characteristics of injury during the FELDA/FAM National Futsal League 2010.

	FELDA/FAM 2010		
League	Total	Men	Women
No. of matches	141	70	71
Injury report forms returned	282 (100%)	140 (100%)	142 (100%)
Player hours	940	466.7	473.3
Player matches	1410	700	710
Incidence of injury per 1000 player matches (95% CI)	61.0 (48.1 to 73.9)	64.3 (45.5 to 83.1)	57.7 (40.0 to 75.4)
Circumstances	N (%)	N (%)	N (%)
Non-contact injuries	32 of 86 (37%)	14 of 45 (31%)	18 of 41 (44%)
Contact injuries	54 of 86 (63%)	31 of 45 (69%)	23 0f 41 (56%)
Contact injuries cause by foul	31 of 54 (57%)	19 of 31 (61%)	12 of 23 (52%)
Estimated severity of injury	N (%)	N (%)	N (%)
0 days	61 (71%)	34 (76%)	27 (66%)
1–7 days	7 (8%)	4 (9%)	3 (7%)
1 week	13 (15%)	6 (13%)	7 (17%)
>1 month	5 (6%)	1 (2%)	4 (10%)
No. of time loss injuries	25	11	14
Incidence of injury per 1000 player hours (95% CI)	26.6 (16.2 to 37.0)	23.6 (16.7 to 30.5)	29.6 (14.1 to 45.1)
Incidence of injury per 1000 player matches (95% CI)	17.7 (10.8 to 24.6)	15.7 (11.1 to 20.3)	19.7 (9.4 to 30.0)

Note: FELDA  =  Federal Land Development Authority; FAM  =  Football Association of Malaysia.

With relative number of players from different playing position accounted, goalkeepers had higher incidence of injury compared with players in other position (defender and forwards) (117.0 vs. 85.1 injuries per 1000 player hours). The difference found however was not statistically significant (*Z* = 1.30, *p*>0.05).

### Injury types

In this study most injuries were classified as new injury (n = 73; 85%). The commonest injury diagnosed was contusion (n = 31; 36%) followed by ligament sprains and muscle strains (Table 3). Majority of contusions (n = 27; 87.1%) did not result in time-loss from participation, whereas around 42.9% (n = 18) of sprains and strains injuries did resulted in time-loss to participation.

**Table 3:** Type of injury diagnosed during the 2010 FELDA/FAM National Futsal League.

Type of injury	No. (%) of players
Contusion	31 (36)
Lower limb	18
Upper limb	8
Trunk	3
Head & neck	2
Sprain	28 (33)
Lower limb	22
Upper limb	6
Strain	14 (16)
Calf	5
Thigh	6
Back	2
Forearm	1
Fracture	7 (8)
Lower limb	4
Upper limb	3
Concussion	3 (4)
Shoulder dislocation	1 (1)
Others	2 (2)

Note: FELDA  =  Federal Land Development Authority; FAM  =  Football Association of Malaysia.

### Injury circumstances

More than half (n = 54; 63%) of injuries resulted from contact with another player (n = 50; 59%), the futsal ball (n = 2; 2%) or the goalpost (n = 2; 2%). Most (57%) contact injuries or 42% of all injuries were caused by foul play as determined by the referee. Fortunately, only eight (22%) injuries rising from a foul play resulted in time-loss to participation. Majority of injuries (71%) did not lead to any time-loss from participation.

### Injury locations

Injuries often affected the lower limb, especially the knee (n = 20; 23%) and ankle (n = 18; 21%). This was followed by injuries to the trunk (n = 12; 14%) and the upper limb (n = 11; 13%). Injury to the head occurred less often (n = 7; 8%). Of the total seven head injuries, concussions were diagnosed in three women players (3.2 per 1000 player hours). The remaining were minor head injuries to the eyes and lips

### Injury severity

The incidence of time-loss injuries was 26.6 per 1000 player hours (95% CI: 16.2 to 37.0), or 17.7 (95% CI: 10.8 to 24.6) per 1000 player matches. Injury incidence from the men's league was compared with the injury data from 2004 and 2008 Men's World Futsal Championship (Table 4).

**Table 4:** Incident and characteristics of injuries during the 2010 FELDA/FAM National Futsal League and World Cups.

	FELDA/FAM 2010 (Men's League)	Brazil 2008 [6]	Chinese Taipei 2004 [6]
No. of matches	70	56	40
Injury report forms returned	140 (100%)	107 (95.5%)	80 (100%)
Matches hours	466.7	373.5	266.7
No. of injuries	45	60	63
Player hours	286.7	356.7	266.7
Incidence of injury per 1000 player hours (95% CI)	96.4 (68.2 to 124.6)	168.5 (125.8 to 211.2)	236.0 (177.8 to 295.2)
Player matches	430	535	200
Incidence of injury per 1000 player matches (95% CI)	64.3 (45.5 to 83.1)	111 (83.0 to 149.0)	158 (119.0 to 197.0)
Circumstances	N (%)	N (%)	N (%)
Non-contact injuries	14 of 45 (31%)	28 of 58 (48%)	19 of 58 (33%)
Contact injuries	31 of 45 (69%)	30 0f 58 (52%)	39 of 58 (67%)
Contact injuries cause by foul	19 of 45 (42%)	4 of 27 (15%)	25 of 39 (64%)
Estimated severity of injury	N (%)	N (%)	N (%)
0 days	34 (76%)	21 (41%)	29 (64%)
1–7 days	4 (9%)	23 (43%)	14 (31%)
1 week	6 (13%)	8 (15%)	2 (3%)
>1 month	1 (2%)	1 (2%)	-
Time loss injuries	11	32*	18**
Per 1000 player hours (95% CI)	23.6 (16.7 to 30.5)	≥89.9 (58.8 to 121.0)	≥67.5 (22.3 to 98.7)
Per 1000 player matches (95% CI)	15.7 (11.1 to 20.3)	≥60 (39.2 to 80.8)	≥45 (24.2 to 65.8)

Note: * Information on time loss from sport is missing in 7 injuries.

** Information on time loss from sport is missing in 18 injuries.

FELDA  =  Federal Land Development Authority; FAM  =  Football Association of Malaysia; N/A  =  not available.

A significant association between time-loss injury and match surface were found, *χ* ^2^ (1,N = 86) = 3.99, *p* = 0.04. Time-loss injuries often (n = 17; 68%) occurred when injuries were sustained during matches played on vinyl surface. Further, significant association between time-loss injury and type of injury was observed, *χ* ^2^ (1,N = 86) = 10.11, *p* = 0.018. Ligament sprains (knees and ankles) were the most frequent injuries (n = 14; 56%) leading to time-loss from participation.

No significant association between age, playing experience, gender, ethnicity, stages of tournament, playing positions, and region of injury, with time-loss injury was demonstrated (*p*>0.05)(Table 5).

**Table 5:** Factors associated with injury severity during the 2010 FELDA/FAM National Futsal League.

Characteristics	Severity of injury		Mann Whitney U†/Chi square test‡	
	Time-loss (N = 25)	No time-loss (N = 61)	*z*/χ^2^	*P* value
Age†, median±IQR (years)	25.0±6.3	23.0±5.8	−0.81	0.421
Experience†, median±IQR (years)	3.0±2.0	3.0±2.0	−0.88	0.382
Sex‡, n (%)			0.98	0.322
Men	11 (44.0)	34 (55.7)		
Women	14 (56.0)	27 (44.3)		
Ethnicity‡, n (%)			0.03	0.854
Malay	24 (96.0)	58 (95.1)		
Others	1 (4.0)	3 (4.9)		
Venus surfaces‡, n (%)			3.99	0.046*
Vinyl	17 (68.0)	27 (44.3)		
Wood	8 (32.0)	34 (55.7)		
Tournament stage			4.47	0.050
Preliminary	20 (80.0)	34 (55.7)		
Final	5 (20.0)	27 (44.3)		
Nature of injury‡, n (%)			10.11	0.018*
Sprain	14 (56.0)	14 (23.0)		
Strain	4 (16.0)	10 (16.4)		
Contusion	4 (16.0)	27 (44.2)		
Others	3 (12.0)	10 (16.4)		
Player's position‡, n (%)			0.72	0.697
Goalkeeper	5 (20.0)	17 (27.9)		
Defender	5 (20.0)	13 (21.3)		
Forward	15 (60.0)	31 (50.8)		

Note: FELDA  =  Federal Land Development Authority; FAM  =  Football Association of Malaysia.

* *p* < 0.05.

† Mann Whitney U test.

‡ Chi square test.

## Discussion

This the first prospective study conducted in Malaysia to look into the incidence and pattern of injuries among futsal player during a local tournament. The primary objective was to explore on futsal related injuries among amateur men and women futsal players. The tournament in the present study was comparatively longer in duration (1^st^ July until 28^th^ November 2010) compared with other studies [5]–[7], [12].

As the numbers of non-goalkeepers exceeded goalkeepers in each game (ratio of 2 to 8), it was not surprising to observed greater proportion of injuries occurred among non-goalkeepers. However when relative numbers of players in different playing positions were considered, goalkeepers had higher injury rates compared with non-goalkeepers. The difference found however was not statistically significant. This finding was consistent to those reported by Lindenfeld et al. (1994) and Putukian et al. (1996). Both studies also reported comparable injury rates between goalkeepers and non-goalkeepers [10], [13].

Most injury resulted from player-to-player contact. Injuries from player-to-ball and player-to-goalposts contact occur less often. This finding was comparable to those reported during Futsal World Cup 2004 and 2008 [6]. Not surprisingly, contusion was the most frequent injury diagnosed throughout the tournament. Faster speed of movement in a smaller playing field may increase the likelihood of collision [4], [5]. Also, more than half of the contact injuries observed were caused by foul play. Fortunately, these contusion injuries were mild and most did not lead to any time-loss from participation. Similar findings were reported by other researchers [6], [7], [13]. In contrary higher proportion of sprains and strains injury were associated with time loss from participation. This finding was consistent with those observed in earlier study [6].

The body region most often injured was the lower limb, specifically the knees and ankles, followed by injuries to the trunk and the upper limbs. As futsal is a fast paced game relying mostly on the lower extremity for ball control, sprinting and frequent changes in direction such observations were anticipated. Similar observations were reported during FIFA Futsal World Cups 2000–2008, and the 15^th^ Brazilian Indoor Soccer Sub20 Team Selection Championship [5], [7], [13].

Interestingly, from the total of seven head injury, only three concussions were diagnosed. The incidence of concussion (3.2 per 1000 player hours) was lower compared with previous Futsal World Cups 2000 to 2008 combined (8.3 per 1000 player hours) [6]. In this study, all concussions resulted from contact injuries. Interestingly concussions were diagnosed only among women futsal players.

The overall incidence of injuries in the FELDA/FAM Men's League was lower when compared with incidence reported in the 2008 and 2004 Men's Futsal World Cups (96.4 vs. 168.5 injuries per 1000 player hours)[6]. In addition the rate of time-loss injuries was also lower (23.6 vs. 89.9). Lower injury rates noted in the current study could be attributed to the difference in playing level. van Beijsterveldt et al. (2014) suggested increase match intensity at higher competition level could explain greater injury incidence among professional players [14]. Another possible explanation for lower injury incidence could be attributed to longer duration of competition (141 vs. 14–16 days). Longer breaks between matches could provide athletes enough time to recover and reduced accumulation of fatigue and reduce risk of injury [15].

Interestingly the incidence of contact injuries cause by foul has dropped from 64% in Futsal World Cup 2004 to 15% in Futsal World Cup 2008. Lower incidence in 2008 could be attributed to stricter rules on fouls and misconduct in the updated futsal laws 2008 (law 11) [16].

No significant difference in injury rates between men and women was found. Injury incidences were 96.4 and 86.6 per 1000 player hours in the men and women's leagues respectively (*Z* = 0.50, *p*>0.05). Similar findings were reported by other researchers [10], [13]. In a 7-week injury surveillance study at a local indoor soccer arena, the overall injury incidence was equal among men and women (5.04 and 5.03 injuries per 100 player hours) [10]. During the Soccer America Dawn to Dark Indoor Soccer Tournament, a higher injury incidence was reported among men (5.79 versus 4.74 per 100 player hours); however, this difference was not statistically significant. It is reminded that these studies only considered injuries that lead to time lost from practice or matches [13]. Minor injuries without time-loss were not included in their analysis.

A significant association between playing surface and time-loss injury was found. Although the number of injuries occurred between the two surfaces was comparable (44 vs. 42), time-loss injuries occurred often when matches were held in vinyl surface. Olsen et al (2004), using videography examination of injury mechanism for anterior cruciate ligament (ACL) injuries in handball, noted more injuries occurred on synthetic, rubberized indoor floors than on wooden floors. They speculated higher friction between the shoe and the vinyl floors may be responsible for higher number of injuries observed [17]. The current study also found a significant association between time-loss injuries and ligament sprains. This observation was consistent with those reported by earlier study [6].

This study was the first cohort study to explore on injury incidence and characteristics during a futsal tournament in Malaysia. The overall incidence of injury noted during the 2010 FELDA/FAM National Futsal Tournament was lower compared with previous study [6]. It is comforting to know that most injuries were minor and did not lead to time-loss from participation.

### Limitations

This study has several limitations. First, data was available only for players who have suffered injuries. Logistics reasons has prevented access to information of all players competed in the tournament. Therefore comparisons cannot be made between injured and non-injured players. Second, injury data were collected only during competition. Injuries that occurred at training or practice sessions were not recorded. Finally, data recording and analysis were performed only on injuries reported to the tournament medical team, possibly other minor injuries that were self-treated by the players or coaches may have been missed.

Despite these limitations, this study has provided important information on futsal related injuries among amateur players (men and women), which can be used to guide future studies.

## Conclusion

Lower injury rate was noted during the 2010 FELDA/FAM National Futsal Tournament. Most injuries were minor resulted from contact with another player. Injuries often involved the lower extremity specifically the knee and ankles. Players diagnosed with muscle strain are significantly more likely to miss their next training session or a match compared to other injuries. Further, knowledge on injury mechanism in futsal is imperative in developing specific injury-preventive intervention.

## Supporting Information

Figure S1 **Injury report form used in the present study.**
(PDF)
