# Supplementary material for: Incidence and Characteristics of Injuries during the 2010 FELDA/FAM National Futsal League in Malaysia
Source: PLoS One. 2014 Apr 14;9(4):e95158. doi: 10.1371/journal.pone.0095158 (PMC3986338; doi:10.1371/journal.pone.0095158)
Supplement: Figure S1 — Injury report form used in the present study. (PDF) [file pone.0095158.s001.pdf]

Figure S1: Injury report form used in the present study.

SPORTS INJURY REPORTING FORM NFL 2010

SPORTS INJURY REPORTING FORM NFL 2010

Name: ____________________ **Gender: Male / Female** **Jersey No:** ______ **Venue:** ____________________

D.O.B: ____ / ____ / ____ **Position:** ________________ **Team:** ____________________ **Playing experience:** ______________

Date of Injury ___ / ___ / ___

Type of activity at time of injury

- ☐ training/practise
- ☐ competition
- ☐ other ________________

Reason for Presentation

- ☐ new injury
- ☐ exacerbated/aggravated injury
- ☐ recurrent injury
- ☐ illness
- ☐ other________________

Body Region Injured

Circle body parts/injured & name

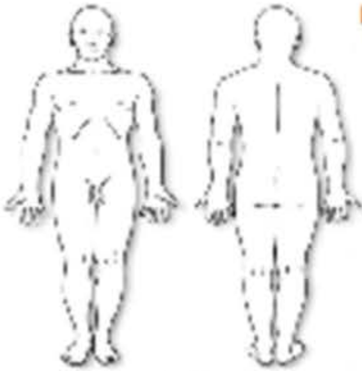

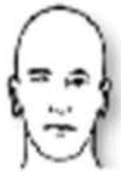

FMARC

- ☐ 1 Head
- ☐ 2 UL
- ☐ 3 Trunk
- ☐ 4 Hip
- ☐ 5 Groin
- ☐ 6 Thigh
- ☐ 7 Knee
- ☐ 8 Leg
- ☐ 9 Ankle
- ☐ 10 Foot

Body part/s

____

____

Nature of Injury/Illness

- ☐ sprain eg ligament tear
- ☐ strain eg muscle tear
- ☐ bruise/ contusion
- ☐ open wound/laceration/cut
- ☐ fracture (including suspected)
- ☐ dislocation/ subluxation
- ☐ abrasion/ graze
- ☐ blister
- ☐ concussion
- ☐ other ________________
- ☐ inflammation/ swelling
- ☐ overuse injury to muscle or tendon
- ☐ cardiac problem
- ☐ respiratory problem
- ☐ loss of consciousness
- ☐ unspecified medical condition

Provisional diagnosis/es __________

____

____

CAUSE OF INJURY

Mechanism of Injury

- ☐ struck by other player
- ☐ struck by ball or object
- ☐ collision with other player/referee
- ☐ collision with fixed object
- ☐ fall/stumble on same level
- ☐ jumping to shoot of defend
- ☐ fall from height/awkward landing
- ☐ twisting to pass or accelerate
- ☐ throwing/ push/ block
- ☐ overexertion (eg muscle tear)
- ☐ overuse
- ☐ slip/trip
- ☐ temperature related eg heat stress
- ☐ other ________________

FMARC Explain exactly how to the incident occurred

1 ______________________________
2 ______________________________
3 ______________________________
4 ______________________________
5 ______________________________
6 ______________________________
7 ______________________________
8 ______________________________
9 ______________________________
10 ______________________________

Were there any contributing factors to the incident, unsuitable footwear, playing surface, equipment, foul play?

____

____

____

Protective Equipment

Was protective equipment worn on the injured body part?

Yes ☐ No ☐

If yes, what type eg mouthguard, ankle, brace, taping

____

Initial Treatment

- ☐ none given (not required)
- ☐ RICE
- ☐ dressing
- ☐ sling, splint
- ☐ crutches
- ☐ massage
- ☐ physiotherapy
- ☐ CPR
- ☐ stretch/exercises
- ☐ strapping/taping only
- ☐ none given - referred elsewhere
- ☐ analgesic
- ☐ other ________________

Advice Given

- ☐ immediate return unrestricted activity
- ☐ able to return with restriction
- ☐ unable to return at present time

Referral

- ☐ no referral
- ☐ medical practitioner
- ☐ physiotherapist
- ☐ other professional
- ☐ hospital
- ☐ other ________________

Provisional severity assessment

- ☐ none (0 day)
- ☐ mild (1-7 days modified activity)
- ☐ moderate (> 1 week modified activity)
- ☐ severe (> 1 month modified or lost)

Treating person

- ☐ medical practitioner
- ☐ physiotherapist
- ☐ paramedic (nurse, medical assistant)
- ☐ sports trainer
- ☐ other ________________

Name of treating person

____

____

Today's Date : ___ / ___ / ___

Specific recommendations : __________

____

____

____
